# Modeling the relationship between body weight and energy intake: A molecular diffusion-based approach

**DOI:** 10.1186/1745-6150-7-19

**Published:** 2012-06-29

**Authors:** Zhejun Gong, Zhefeng Gong

**Affiliations:** 1 College of Logistics Engineering, Wuhan University of Technology, Wuhan, Hubei Province, 430063, China; 2 School of Medicine, Zhejiang University, Hangzhou, Zhejiang, 310058, China

**Keywords:** Molecular diffusion, Body weight, Model, Choice making

## Abstract

**Background:**

Body weight is at least partly controlled by the choices made by a human in response to external stimuli. Changes in body weight are mainly caused by energy intake. By analyzing the mechanisms involved in food intake, we considered that molecular diffusion plays an important role in body weight changes. We propose a model based on Fick's second law of diffusion to simulate the relationship between energy intake and body weight.

**Results:**

This model was applied to food intake and body weight data recorded in humans; the model showed a good fit to the experimental data. This model was also effective in predicting future body weight.

**Conclusions:**

In conclusion, this model based on molecular diffusion provides a new insight into the body weight mechanisms.

**Reviewers:**

This article was reviewed by Dr. Cabral Balreira (nominated by Dr. Peter Olofsson), Prof. Yang Kuang and Dr. Chao Chen.

## Background

Body weight change is a complex behavioral response associated with appetite regulation and energy metabolism [1]. Although changes in body weight involve genetic, metabolic, biochemical, cultural and psychosocial factors, the two main factors that regulate body weight are food intake and energy expenditure [2,3]. In recent years, mathematical models have become increasingly used in medical research. These models have helped researchers to develop new ways of dealing with animal behaviors. In terms of body weight, behavioral economic models have been developed to address the effects of environmental factors on energy intake and body weight [4]. A series of experimental studies have also been conducted to develop mathematical models to describe the physiological basis of body weight. In fact, these models can quantitatively address the metabolic processes underlying body weight changes and can be used to aid body weight control [5-8]. A mathematical model has also been proposed to address the molecular mechanisms underlying body weight, although the validity of the model has not been verified experimentally [9].

In this paper, we examined the impacts of energy intake and energy expenditure on body weight. Neuropeptides are small protein-like molecules released by neurons to communicate with each other. These neuronal signaling molecules influence specific activities of the brain, including control of food intake [1,10-12]. Neuropeptides are expressed and released by neurons, and mediate or modulate neuronal communication by acting on cell surface receptors. They have a long half-life, show high affinity for their receptors, and reach their target by diffusion, often over a long distance [13-15]. More specifically, food intake can induce the synthesis of specific neuropeptides that diffuse to activate metabolic processes [10]. Considering the above discussion on the neural regulation of obesity, it seems likely that the molecular mobility (diffusion) of neuropeptides, for example, plays an important role in body weight regulation. In other words, the body converts food stimuli to molecular signaling processes. The molecular mobility of body weight control is at least partly explained by the diffusion of molecules inside or outside of neural cells. Accordingly, changes in body weight are influenced by molecular movements driven by energy intake. Fick’s second law, also known as the diffusion equation, describes non-steady-state diffusion, and is typically used to model molecular mobility [16]. Therefore, we can use the molecular diffusion model to describe body weight behavior, replacing molecular concentration with calorie intake as the driving force in this process. It is known that some biological molecules are synthesized at high concentrations and subsequently affect the concentrations of other molecules by diffusion, until the resulting behavior is established. Therefore, we incorporated the diffusion equation as a model of body weight control and validated this model using experimental data. Because the diffusion equation is nonlinear, the correct parameters can be obtained by global optimization.

In summary, we propose a model in which body weight control is derived from molecular diffusion. We also quantitatively investigate the relationship between energy intake and body weight, by applying Fick’s second law of diffusion in combination with a mathematical algorithm. Validation of the model with experimental data obtained from humans showed that the model dynamically simulates changes in body weight and energy intake very well. This model is suitable for describing the relationship between energy intake and body weight.

## Results and discussion

### Body weight change: a molecular diffusion based process

Because molecular mobility is accompanied by energy transference, we can describe molecular diffusion with energy diffusion. The human body obeys the law of energy conservation [7], which can be expressed as

(1) $$\frac{d}{dt}(\rho *V)=\frac{dE}{dt}$$

where *ρ* is the energy density of body mass, *V* is the body mass, *E* is the net energy intake, *t* is the time.

Suppose *J* is energy flux (amount of energy per unit area per unit time in direction *x*), p is the energy density of body fat mass. For healthy adults (18-59y), body weight changes largely due to fat mass (FM) [17], so d(ρ*V) is approximately equal to p*dV. We have

(2) $$\frac{dE}{dt}=-\frac{dJ}{dx}$$

and

(3) $$J=-D\frac{pdV}{dx}$$

where *D* is energy diffusion coefficient. Substituting Equation 2 and Equation 3 into Equation 1 leads to the following equation:

(4) $$\frac{dV}{dt}=D\frac{d^{2}V}{dx^{2}}$$

Equation 4 is actually the form of Fick’s second law of diffusion.

In the initial conditions where *t* = 0 and *x* > 0, then *V* = *V*_*0*_. In marginal conditions where *t* > 0 and *x* = 0, then *V* = *V*_*s*_*.* When *t* > 0 and *x* = ∞, *V* = *V*_*0*_. *V*_*0*_ is the initial body mass, *V*_*s*_ is the body mass transformed from energy intake. Therefore, the solution of Equation 4 is:

(5) $$\begin{array}{ll} V(x,t) & =V_{s}[1-erf(x/(2\sqrt{Dt}))]\\ & +V_{0}erf(x/(2\sqrt{Dt})) \end{array}$$

where $erf(c)=(2/\sqrt{\pi })\int_{0}^{c}\exp(-c^{2})dc$. Because $V_{s}=E/p$, Equation 5 can be rewritten as the following equation:

(6) $$\begin{array}{ll} V(t) & =\frac{1}{p}E[1-erf(x/(2\sqrt{Dt}))]\\ & +V_{0}erf(x/(2\sqrt{Dt})) \end{array}$$

From the above discussion, we can know the body weight change process is a diffusion process driven by energy intake.

### Fitting and the model to experimental data and validation

As described above, changes in body weight can be explained by molecular movement driven by energy intake. Considering that body weight change mimics molecular diffusion, and that diffusive processes are involved in body weight changes at the cellular level, this behavioral activity can be described by Equation 6.

To use the molecular diffusion based model to describe the relationship between energy intake and body weight, because distance *x* represents body attributes, it is set as a constant in this model. In this way, Equation 6 can be rewritten as:

(7) $$f(t)=b*erf(\beta /\sqrt{t})+\alpha *l*[1-erf(\beta /\sqrt{t})]$$

where *f(t)* = body weight, *b* = initial body weight, *l* = energy intake, *t* = time of feeding, and *α* and *β* are constants. If *t* > 1, this formula can be rewritten as follows:

(8) f(t)=f(t−1)*erf(β)+α*l(t)*[1−erf(β)]

where *l(t)* = energy intake, with other parameters identical to those in Equation 7.

Equation 8 can then be applied to simulate experimental data and its validity tested against reference data (in this case human body weight). To best estimate the model parameters, ISCEM algorithm was adopted because this algorithm can not only estimate parameters in complex functions but also conduct global optimization [18]. Energy intake and body weight were recorded for humans in an earlier study [19]. If the experimental data and model-derived data show a good fit, we can conclude that the model is suitable to describe the relationship between energy intake and body weight.

### Simulation of body weight change using the developed model

Using the experimental data recorded over 24 weeks (Table 1) and the ISCEM algorithm, the following constants were obtained:

**Table 1:** Group’s body weight related data (S1-S24) from Minnesota human starvation study

**Time (week)**	**Body weight (kg)**	**TEE (kcal/day)**	**Mean energy intake (kcal/day)**	**Net energy intake (kcal/day)**
0	69.39	1934.33	3538.72	1604.39
S1	68.35	1884.53	1658	−226.53
S2	66.8	1835.21	1658	−177.21
S3	65.76	1786.36	1648.88	−137.48
S4	64.29	1737.6	1610.88	−126.72
S5	63.33	1691.55	1645.94	−45.61
S6	62.16	1643.45	1639.16	−4.29
S7	61.11	1595.75	1639.03	43.28
S8	60.31	1548.28	1634.84	86.56
S9	59.56	1500.8	1620.41	119.61
S10	58.71	1453.31	1595.31	142
S11	58.14	1405.63	1578.72	173.09
S12	57.28	1357.94	1525.16	167.22
S13	56.6	1346.8	1515.69	168.89
S14	56.16	1335.67	1492.84	157.17
S15	55.69	1324.54	1459.94	135.4
S16	54.7	1313.39	1430.5	117.11
S17	54.28	1302.28	1488.81	186.53
S18	54.08	1291.1	1486.44	195.34
S19	53.51	1281.22	1519.72	238.5
S20	53.18	1271.34	1515.47	244.13
S21	52.99	1261.46	1538.75	277.29
S22	52.9	1251.58	1554.06	302.48
S23	52.83	1241.7	1581.19	339.49
S24	52.57	1231.83	1641.63	409.8

*α* = 0.016337, *β* = 1.7096

Entering these constants yields the following equation:

(9) $$\begin{array}{ll} f(t) & =f(t-1)*erf(1.7096)+0.016337*7\\ & *l(t)*[1-erf(1.7096)] \end{array}$$

**Figure 1:**
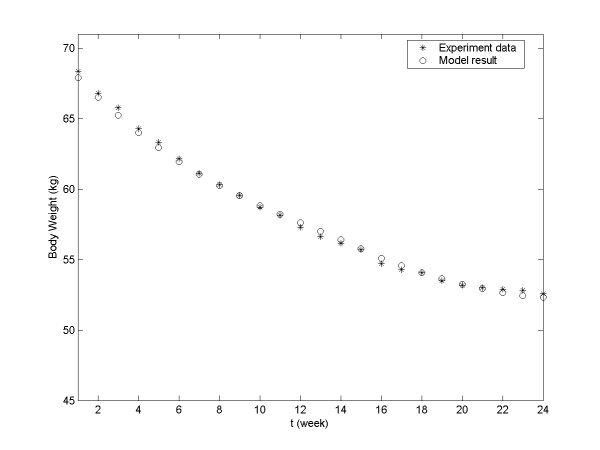
**Comparison of experimentally recorded and model-generated group’s body weight of humans (weeks S1–S24).** Asterisks, experimentally recorded data; circles, model-generated data.

Using Equation 9, we can estimate daily body weight from week S1 to week S24. The model-generated body weight data are plotted alongside the actual experimental data in Figure 1. The determination coefficient (R^2^) for this plot was 0.99666, which indicates that the model-generated data closely match the experimental data. Comparison between the actual experimental body weight and model result of each subject is shown in Appendix A.

### Model validation and body weight prediction

We next sought to validate the model. To achieve this, body weight measured between week S1 and week S12 from Table 1 were entered into the ISCEM algorithm, which yielded the following constants:

*α* = 0.0170757, *β* = 1.7029

Entering these constants into Equation 8 yields the following equation:

(10) $$\begin{array}{ll} f(t) & =f(t-1)*erf(1.7029)+0.0170757*7\\ & *l(t)*[1-erf(1.7029)] \end{array}$$

**Figure 2:**
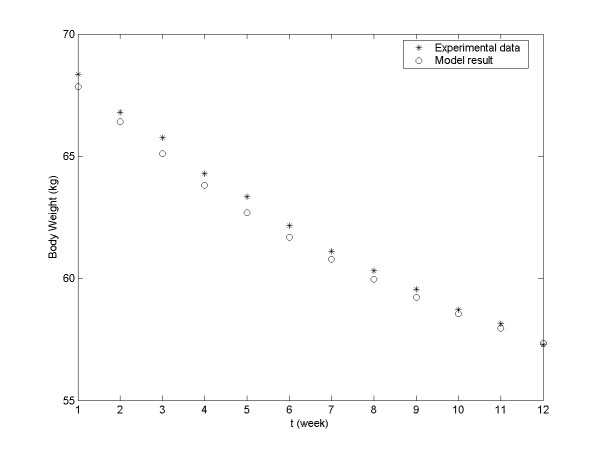
**Comparison of experimentally recorded and model-generated group’s body weight of humans (weeks S1–S12).** Asterisks, experimentally recorded data; circles, model-generated data.

Using Equation 10, it is possible to estimate the daily body weight from week S1 to week S12. The model-generated data are plotted alongside the experimental data in Figure 2. R^2^ for this model was 0.98499, indicating very close fit between the model and the experimental data.

**Figure 3:**
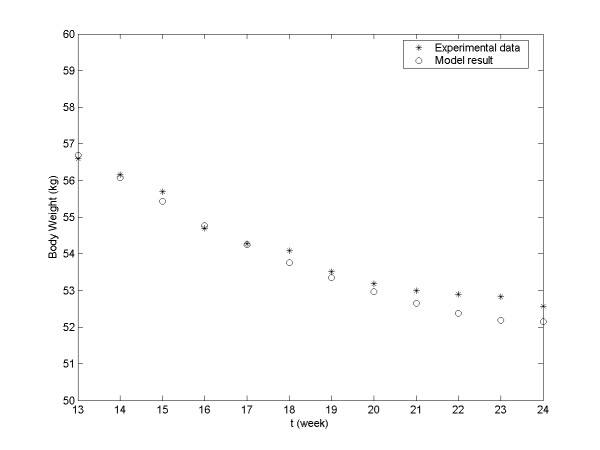
**Prediction of group’s body weight during weeks S13–S24 based on actual experimental data from weeks S1–S12.** The actual experimental data in weeks S13–S24 are also shown. Asterisks, actual experimental data; circles, model-generated data.

Finally, we used this model with the parameters based on the experimental data for weeks S1–S12 to predict body weight change between week S13 and week S24. The body weights predicted for weeks S13–S24 and the corresponding experimental data are plotted in Figure 3. The R^2^ for this model was 0.94229, indicating the model satisfactorily fits the experimental data. Confidence intervals for predicted body weight of each subject were provided in Appendix B.

## Methods

### Ethics statement

Because human data were used, approval was obtained from Wuhan University of Technology's Ethics Committee. This research was based on experimental data from literature [19]. As such, no consent statement for participation is required.

### The Minnesota starvation study

The study reduced the energy intake of 32 male conscientious objectors (20–33 y old, mean 25.5 y) to decrease body mass comparably to severely undernourished prisoners of war with the aim of testing methods for rehabilitating starved men. The study included a 12-week control phase (weeks C1–C12), 24 weeks of energy restriction (weeks S1–S24), and 20 weeks of recovery (R1–R20). During weeks C1–C12, energy intake was adjusted to bring individuals towards the group norm, based on weight for height, with a mean weight loss of 0.80 kg. Physical activity included 22 miles per week of outdoor walking and additional walking on campus, plus custodial duties. All subjects were also required to walk at 3.5 miles per hr for half an hr per week on a motor-driven treadmill with a 10% grade. The control diet contained about 100 g of protein, 400 g of carbohydrates, and 130 g of fat. Energy intake averaged 3,492 kcal/d (14.62 mJ/d) for the last 3 control weeks, during which group weight declined only 0.3 kg. From then on, subjects were fed at a level that was expected to cause a 24% group average decrease in body mass during the next 24 weeks. Weight loss was induced by reducing food intake to two daily meals with 51 g of protein, 286 g of carbohydrates, and 30 g of fat, with 3 basic menus consisting of cereal, whole-wheat bread, potatoes, turnips, and cabbage, supplemented by scant amounts of meat and dairy products. During the entire starvation period, walking 22 miles a week and custodial work remained mandatory [19].

Total Energy Expenditure (TEE) includes two major parts: Resting Energy Expenditure (REE), the amount of calories needed to maintain basic body systems and body temperature at rest; Activity Energy Expenditure (AEE), the amount of calories used during activity [20]. Net energy intake is the difference between food intake and TEE. Although TEE was not measured in the Minnesota starvation study, TEE can be obtained through calculating REE and AEE [19,21].

Some useful data are shown in Table 1.

### ISCEM algorithm: an improved SCEM-UA algorithm

The shuffled complex evolution metropolis algorithm (SCEM-UA) is a global-searching algorithm developed by Vrugt JA et al. [22]. The SCEM–UA method adopts Markov Chain Monte Carlo theory (MCMC) and uses the Metropolis–Hastings algorithm (MH), replacing the Downhill Simplex method, to obtain a global optimal estimation. Although SCEM-UA can successfully obtain the global optimal solution, its performance depends on correct setting of the minimal and maximal limits. In the current study, we improve the SCEM-UA algorithm so that it can optimize the parameter searching space and obtain the optimal solution. This improved algorithm is termed the ISCEM algorithm.

Suppose *ŷ* = *η*(*ξ*|*θ*), where *ŷ* is an *N* × 1 vector of model predictions, *ξ* is an *N* × *n* matrix of input variables and *θ* is a vector of *n* unknown parameters. The SCEM-UA algorithm is given below:

(1)  To initialize the process, choose the population size *s* and the number of complexes *q*. The algorithm tentatively assumes that the number of sequences is identical to the number of complexes.

(2) Generate *s* samples from the prior distribution {*θ*_*1*_*θ*_*2*_,…,*θ*_*s*_} and compute the posterior density {*p*(*θ*^(1)^|**y**),***p***(*θ*^(2)^|**y)**,…,*p*(*θ*^(s)^|**y)**} at each point [22].

(3) Sort the points in order of decreasing posterior density and store them in an array D[1:*s*,1:*n* + 1], where *n* is the number of parameters, so that the first row of D represents the point with the highest posterior density. The extra column stores the posterior density. Initialize the starting points of the parallel sequences, S^1^,S^2^,…,S^*q*^, such that S^*k*^ is D[*k*,1:n + 1], where *k* = 1,2,…,*q*.

(4) Partition D into *q* complexes C^l^,C^2^,…,C^*q*^, each containing *m* points, such that the first complex contains every *q*(*j* − 1) + 1 ranked point, the second complex contains every q(j−1)+2 ranked point of D, and so on, where *j* = 1,2,…,*m*.

(5) Initialize L,T,AR_min_, c_n_. For each C^*k*^, call the SEM algorithm [22] and run it L times;

(6) Unpack all complexes C back into D and rank the points in order of decreasing posterior density.

(7) Check Gelman and Rubin (GR) convergence statistic. If convergence criteria are satisfied, stop; otherwise, return to step 4.

The ISCEM algorithm is given below:

1) Suppose I_min_≤*θ*≤I_max_, I_min_ and I_max_ are interval vectors of *θ*. The initial I_max_ is set to be very large. Run the SCEM-UA algorithm and let the output parameter vector with highest posterior density (*p*_*o*_) be *θ*_*o*_. Set I_max_ = *θ*_*o*_.

2) Run the SCEM-UA algorithm again, and let the output parameter vector with highest posterior density (*p*_*w*_) be *θ*_*w*_. If || *p*_*o*_ - *p*_*w*_ || ≤ *ε*, where *ε* > 0, go to step (4); otherwise set *θ*_*o*_ = *θ*_*w*_.

3) If *p*_*o*_ ≤ *p*_*w*_, let I_max_ = *θ*_*w*_; otherwise, let I_min_ = *θ*_*w*_ . Let *p*_*o*_ = *p*_*w*_, go to step (2).

4) Output *θ*_*w*_ .

## Conclusions

In this paper, we have shown that energy intake and energy expenditure in humans can be simulated using a mathematical algorithm based on molecular diffusion. In the model, only the effects of calorie intake on body weight are considered; other variables that may affect body weight are included as constants. This is because the internal and external environmental factors that may influence body weight can be assumed to be stable when environment is stable. In fact, as shown here, if these factors are kept relatively stable, the prediction of body weight based on energy intake and defined constants matches closely with experimental data.

In this model, only the general relationship between energy intake and body weight was examined. We believe this model will provide new insights into the mechanisms underlying body weight control. In future studies, more information is needed to examine the impact of neuronal signaling mechanisms that control body weight on this model.

## Appendix

### Appendix A Table 2

**Table 2:** Comparison of experimental data and Model result of each subject

**No.**	**Weight S1 kg**	**Weight S2 kg**	**Weight S3 kg**	**Weight S4 kg**	**Weight S5 kg**	**Weight S6 kg**	**Weight S7 kg**	**Weight S8 kg**	**Weight S9 kg**	**Weight S10 kg**	**Weight S11 kg**	**Weight S12 kg**	**Weight S13 kg**	**Weight S14 kg**	**Weight S15 kg**	**Weight S16 kg**	**Weight S17 kg**	**Weight S18 kg**	**Weight S19 kg**	**Weight S20 kg**	**Weight S21 kg**	**Weight S22 kg**	**Weight S23 kg**	**Weight S24 kg**	**R**^**2**^
122(p)	64.6	63	61.6	60.2	59	57.6	56.6	55.5	54.6	53.5	52.8	52.1	51.7	51.5	51	50	49.4	49	48.5	48.2	47.7	47.8	47.2	47.4	
122(m)	63.974	62.658	61.434	60.249	59.226	58.294	57.461	56.718	56.046	55.424	54.867	54.309	53.763	53.204	52.615	52.002	51.523	51.067	50.696	50.34	50.049	49.808	49.636	49.593	0.891
123(p)	63.8	62.6	61.8	60.6	60.1	59.1	58.2	57.6	57.3	56.8	56.5	55.8	55.2	54.9	54.8	53.9	53.4	53	52	51.9	51.5	52.1	52.2	52.1	
123(m)	63.285	61.98	60.767	59.591	58.579	57.657	56.834	56.101	55.438	54.826	54.279	53.73	53.192	52.642	52.062	51.458	50.988	50.54	50.177	49.829	49.546	49.313	49.149	49.113	0.685
119(p)	65.5	64.1	63	61.5	60.7	59.4	58.4	57.6	56.9	55.9	55.4	54.5	53.9	53.4	53.2	52.2	51.4	51	50.8	50.5	50.3	50.7	49.8	49.1	
119(m)	64.86	63.531	62.293	61.094	60.058	59.113	58.267	57.511	56.827	56.193	55.624	55.054	54.496	53.926	53.325	52.702	52.212	51.745	51.363	50.997	50.696	50.444	50.263	50.21	0.988
120(p)	69.6	68.2	67.1	65.6	64.4	63.2	61.9	61.1	60.5	59.7	58.7	57.7	56.8	56	55.5	54.7	54	53.4	53	52.3	52.1	51.3	51.2	51.6	
120(m)	69.29	67.891	66.585	65.319	64.218	63.207	62.297	61.479	60.732	60.037	59.409	58.78	58.163	57.536	56.879	56.2	55.655	55.135	54.7	54.282	53.929	53.627	53.396	53.294	0.947
129(p)	64.7	63.3	62.4	61	60.3	59.6	58.6	58.1	57.7	57	57	56.1	55.8	55.5	54.8	53.8	53.4	53.4	53.3	53.2	53	52.8	52.8	52.2	
129(m)	64.171	62.852	61.625	60.436	59.411	58.476	57.64	56.894	56.219	55.595	55.036	54.475	53.926	53.364	52.773	52.158	51.676	51.218	50.844	50.486	50.193	49.949	49.776	49.73	0.731
130(p)	64.8	63.4	63	61.5	60.7	60.1	59	58.5	58	58.1	57.8	57.5	56.9	56.6	56.6	55.4	55.7	55.7	55.7	55.6	54.2	53.9	54.5	53.6	
130(m)	64.565	63.24	62.007	60.812	59.781	58.84	57.998	57.247	56.566	55.937	55.372	54.806	54.252	53.685	53.089	52.469	51.982	51.519	51.141	50.778	50.48	50.232	50.054	50.004	0.104
126(p)	82.6	81.2	79.8	78.1	77.1	75.4	74.1	72.8	71.8	70.6	69.8	69.1	68.1	67.4	67.6	66	65.7	65.4	65.3	63	62	62.6	61.6	60.6	
126(m)	81.89	80.294	78.795	77.338	76.049	74.853	73.762	72.764	71.842	70.973	70.174	69.377	68.595	67.804	66.987	66.15	65.45	64.777	64.191	63.625	63.126	62.681	62.308	62.067	0.988
127(p)	63.1	61.2	60.2	58.3	57.4	56.1	55.5	54.6	54.2	53.2	52.9	52.5	51.8	51.3	51.2	50.8	50.4	49.6	49.2	48.7	49.2	48.7	49.2	49.3	
127(m)	62.793	61.496	60.29	59.122	58.117	57.202	56.386	55.66	55.004	54.399	53.858	53.316	52.785	52.241	51.667	51.069	50.605	50.164	49.806	49.464	49.187	48.959	48.801	48.771	0.971
22(p)	64.2	62.8	61.4	60.2	59.2	58.1	57.2	56.8	56.2	55.4	55	53.8	53.4	53	52.4	51.2	51.2	51.3	50.6	50.5	50	49.4	49.9	49.4	
22(m)	63.679	62.368	61.148	59.967	58.949	58.021	57.192	56.453	55.785	55.168	54.615	54.061	53.518	52.963	52.378	51.769	51.294	50.841	50.473	50.121	49.834	49.596	49.427	49.387	0.995
23(p)	68.3	66.6	65.4	64	62.8	61.6	60.4	59.6	58.5	57.6	56.9	55.8	55	54.6	53.9	53.4	52.8	52.7	52.2	51.8	51.5	51.4	51.4	51.4	
23(m)	67.715	66.341	65.059	63.817	62.739	61.751	60.864	60.068	59.344	58.67	58.063	57.455	56.86	56.252	55.616	54.956	54.431	53.93	53.514	53.114	52.78	52.496	52.282	52.197	0.954
19(p)	69.6	68.3	67.6	65.9	64.6	63.7	62.6	61.7	60.8	59.4	58.6	57.5	56.8	56.1	55.7	54.3	54	51.5	51.4	51.4	52.5	52.4	52.2	50.4	
19(m)	69.093	67.697	66.395	65.131	64.033	63.025	62.118	61.302	60.559	59.867	59.241	58.614	58	57.375	56.721	56.044	55.502	54.984	54.552	54.136	53.786	53.486	53.257	53.157	0.933
20(p)	63.7	62.5	61.5	60.1	59	58	57.1	56.4	55.5	54.9	54.7	53.9	53	52.8	52.1	50.8	49.8	49.4	49.2	48.4	47.8	48.1	48.2	48	
20(m)	63.285	61.98	60.767	59.591	58.579	57.657	56.834	56.101	55.438	54.826	54.279	53.73	53.192	52.642	52.062	51.458	50.988	50.54	50.177	49.829	49.546	49.313	49.149	49.113	0.974
29(p)	69.7	68.1	67.3	66.5	65.5	65.4	64.5	63	60.3	57.4	55.1	54.5	55.2	55.5	54.1	53.3	54.2	54	52.7	52.3	53	54.2	53.8	53.5	
29(m)	69.585	68.182	66.871	65.601	64.495	63.48	62.566	61.743	60.993	60.294	59.661	59.028	58.408	57.777	57.116	56.433	55.885	55.361	54.922	54.501	54.145	53.839	53.605	53.5	0.863
30(p)	67.1	65.6	64.6	63.1	62.3	61	60.4	59.3	59	58.2	58.6	57.6	57	56.2	56	54.8	54.5	54.2	53.6	53.8	53.9	53.1	53.2	52.4	
30(m)	66.632	65.275	64.01	62.784	61.722	60.75	59.879	59.098	58.389	57.731	57.138	56.545	55.963	55.37	54.747	54.101	53.589	53.101	52.698	52.311	51.989	51.718	51.516	51.444	0.944
26(p)	70.3	69.3	67.7	65.8	65	63.5	62.3	61.7	60.6	59.7	59.3	58.1	57.7	58	56.7	56.5	56	57.2	55.8	55.4	55.4	54.7	53.2	53.1	
26(m)	69.782	68.376	67.062	65.789	64.68	63.662	62.745	61.92	61.166	60.465	59.829	59.194	58.571	57.937	57.274	56.589	56.038	55.512	55.071	54.647	54.289	53.981	53.744	53.637	0.98
27(p)	74.5	73.2	72	69.6	68.4	67	65.5	64.4	63.1	61.9	61.5	60.8	60.3	60	58.8	58.1	57.6	57.4	56.8	56.1	55.8	55.3	55.6	55.7	
27(m)	73.621	72.155	70.782	69.451	68.285	67.21	66.238	65.358	64.551	63.797	63.109	62.423	61.749	61.066	60.354	59.62	59.022	58.45	57.963	57.493	57.091	56.739	56.46	56.31	0.958
4(p)	60.9	59.6	58.6	57.2	56	54.9	53.8	53.1	52.3	51.5	51	50.4	50	49.4	48.6	47.9	48.3	48.3	47.5	47.3	47.1	47.1	47.3	47.4	
4(m)	60.627	59.364	58.191	57.056	56.084	55.2	54.415	53.72	53.095	52.519	52.008	51.494	50.992	50.476	49.93	49.359	48.921	48.506	48.175	47.858	47.606	47.403	47.269	47.263	0.971
5(p)	79.6	77.9	76.4	74.8	73.4	72.2	70.6	69.6	68.2	66.9	65.5	64.6	63.7	62.8	62.3	60.7	60	59.6	58.8	58.6	58.1	57.8	57.2	57.1	
5(m)	79.134	77.581	76.124	74.709	73.461	72.306	71.254	70.296	69.411	68.581	67.819	67.059	66.313	65.558	64.776	63.974	63.308	62.668	62.115	61.581	61.115	60.7	60.359	60.148	0.893
1(p)	75.5	73.8	72.1	70.1	68.8	67.3	66	65.3	64.8	64.3	63.8	64.9	64.4	62.6	60.6	59.2	58.8	57.7	57.3	56.6	56.5	56.6	58.2	57	
1(m)	75.59	74.093	72.69	71.329	70.133	69.03	68.029	67.122	66.287	65.505	64.791	64.078	63.379	62.67	61.933	61.175	60.553	59.956	59.446	58.953	58.528	58.154	57.852	57.681	0.932
2(p)	72.1	70.1	69.1	67.9	67	65.7	64.6	63.5	62.6	61.5	61	60.1	58.8	58.2	58.2	57.1	56.5	56.2	54.6	55.1	55.2	57.2	57.9	55.9	
2(m)	71.948	70.507	69.161	67.854	66.713	65.664	64.715	63.859	63.076	62.344	61.68	61.015	60.364	59.702	59.011	58.299	57.722	57.169	56.702	56.253	55.869	55.537	55.276	55.145	0.956
11(p)	65.7	63.9	62.6	61.6	60	59.1	58.2	57.6	56.7	56	55.3	54.4	54.1	53.5	53.2	52.5	51.8	51.9	50.8	50.3	50	49.5	49.9	49.6	
11(m)	64.958	63.627	62.388	61.188	60.151	59.204	58.356	57.599	56.914	56.278	55.709	55.137	54.578	54.006	53.404	52.78	52.288	51.821	51.437	51.07	50.768	50.515	50.333	50.278	0.989
12(p)	79.7	77.5	75.8	74	73.2	71.5	70.4	70	69.5	68.1	68.1	68.7	67.6	67.8	67.7	66.2	65.2	65.1	64.9	63.8	62.9	61.8	61.6	63.2	
12(m)	79.33	77.775	76.315	74.897	73.646	72.488	71.433	70.472	69.585	68.752	67.987	67.224	66.476	65.719	64.934	64.129	63.461	62.818	62.263	61.727	61.258	60.842	60.498	60.285	0.896
8(p)	63.8	62.8	62.7	61.6	60.7	59.2	58.6	58.2	57.2	57.1	56.2	53.9	50.7	50.2	50.3	50.5	49.9	48.8	47.8	47.7	48.8	48.9	48.3	47.5	
8(m)	63.187	61.883	60.671	59.497	58.487	57.566	56.744	56.012	55.351	54.74	54.195	53.647	53.111	52.562	51.983	51.38	50.911	50.465	50.103	49.756	49.474	49.242	49.079	49.045	0.905
9(p)	71.5	69.6	69.1	68	67.2	66.2	64.6	64.1	63.6	64.1	63	60.4	59.6	59.9	59.6	58	57.3	57.6	57.1	57.5	57.4	57.2	56.6	58.1	
9(m)	71.357	69.926	68.588	67.291	66.159	65.118	64.178	63.33	62.555	61.832	61.175	60.518	59.875	59.221	58.537	57.832	57.262	56.717	56.257	55.815	55.438	55.113	54.858	54.733	0.917
104(p)	66.7	64.7	63.9	63	62.5	61.1	60.2	59.3	58.9	58	57.6	56.6	55.8	54.9	53.8	52.9	52.6	52.3	51.8	51.4	51.1	51.4	51.4	51.6	
104(m)	66.632	65.275	64.01	62.784	61.722	60.75	59.879	59.098	58.389	57.731	57.138	56.545	55.963	55.37	54.747	54.101	53.589	53.101	52.698	52.311	51.989	51.718	51.516	51.444	0.985
105(p)	67.4	66	65.4	63.7	63	61.7	61.3	59.9	59.3	58.1	57.5	56.5	55.7	55	54.7	53.5	52.7	52.6	51.4	50.8	51.5	51.4	51.8	51.8	
105(m)	67.223	65.856	64.582	63.347	62.276	61.296	60.416	59.627	58.91	58.243	57.643	57.041	56.452	55.851	55.221	54.568	54.048	53.553	53.143	52.749	52.42	52.142	51.934	51.855	0.974
101(p)	63.7	62.4	61.6	60.2	59.2	58.2	57.2	56.5	55.8	55	54.5	53.6	53.1	53.3	53.1	51.9	51.6	51.8	51.7	51.4	49.3	48.4	48.4	49.7	
101(m)	63.088	61.786	60.576	59.404	58.394	57.475	56.654	55.924	55.264	54.655	54.111	53.564	53.029	52.482	51.904	51.303	50.835	50.389	50.029	49.683	49.403	49.171	49.01	48.976	0.962
102(p)	67	65.5	64.6	63.4	62.2	61	59.9	59	58	57.5	57.2	56.3	55.5	54.5	55.2	53.7	53.4	53.6	53	52.8	53	51.9	51.8	51.9	
102(m)	66.435	65.081	63.819	62.596	61.537	60.568	59.7	58.922	58.215	57.56	56.97	56.379	55.8	55.209	54.589	53.946	53.436	52.951	52.55	52.165	51.846	51.576	51.377	51.306	0.987
111(p)	62.5	60.6	59.4	58.1	57.2	56	54.9	54.3	53.9	53	52.9	52.1	51.8	51.3	50.9	50.4	50.1	50.1	49.8	49.5	49.4	49.1	49	49.1	
111(m)	61.612	60.333	59.145	57.995	57.008	56.11	55.311	54.602	53.963	53.373	52.849	52.322	51.807	51.278	50.719	50.137	49.687	49.26	48.916	48.588	48.325	48.11	47.965	47.948	0.976
112(p)	60.6	58.9	58	56.3	55.9	54.8	53.4	52.5	51.9	51.4	50.8	50.5	50.4	50.7	50.2	48.9	49	50.3	50.6	50.9	50.9	50.4	49	49	
112(m)	60.332	59.073	57.905	56.774	55.806	54.927	54.147	53.456	52.834	52.263	51.756	51.246	50.747	50.236	49.693	49.126	48.692	48.28	47.952	47.639	47.391	47.191	47.06	47.057	0.776
108(p)	66	64.6	63.5	62	61.4	60.6	59.8	59.8	60.5	60	59.1	57.4	56.5	55.8	55.7	55.5	55	55.1	54.4	54.6	55.1	56.6	57.1	54.1	
108(m)	65.451	64.112	62.865	61.657	60.613	59.658	58.804	58.04	57.347	56.705	56.129	55.551	54.985	54.407	53.799	53.168	52.671	52.197	51.808	51.435	51.127	50.869	50.681	50.621	0.324
109(p)	78.3	76.3	75	73.4	72.5	70.9	69.6	68.6	67.8	66.9	66.3	65.4	64.8	64.5	63.7	62.2	61.4	61.5	60.8	60.2	59.6	58.9	59.2	59.5	
109(m)	77.657	76.128	74.693	73.3	72.074	70.941	69.91	68.973	68.11	67.3	66.558	65.817	65.091	64.355	63.592	62.808	62.16	61.538	61.003	60.486	60.037	59.639	59.314	59.12	0.996

*Note: 1) No. is subject number, 122(p) is actual experimental body weight value of subject 122, 122(m) is model result of subject 122,* etc.

*2) R*^*2*^*is determination coefficient.*

3)The experimental data of Subject 130 is not fulfilled. It shows this subject is a special case.

### Appendix B Table 3

**Table 3:** Confidence interval of estimation (Confidence level is 95%)

**Week**	**Weight(kg)**	**Weight(kg)**	**Weight(kg)**	**Weight(kg)**	**Weight(kg)**	**Weight(kg)**	**Weight(kg)**	**Weight(kg)**	**Weight(kg)**	**Weight(kg)**	**Weight(kg)**	**Weight(kg)**
	**S13**	**S14**	**S15**	**S16**	**S17**	**S18**	**S19**	**S20**	**S21**	**S22**	**S23**	**S24**
Subject No.												
122(p)	51.7	51.5	51	50	49.4	49	48.5	48.2	47.7	47.8	47.2	47.4
122(model)	51.588 ± 2.592	51.063 ± 2.443	50.504 ± 2.332	49.919 ± 2.241	49.476 ± 2.145	49.057 ± 2.059	48.728 ± 1.983	48.414 ± 1.919	48.17 ± 1.86	47.977 ± 1.817	47.858 ± 1.767	47.876 ± 1.745
123(p)	55.2	54.9	54.8	53.9	53.4	53	52	51.9	51.5	52.1	52.2	52.1
123(model)	55.229 ± 4.239	54.645 ± 3.992	54.029 ± 3.788	53.387 ± 3.637	52.889 ± 3.493	52.415 ± 3.364	52.032 ± 3.255	51.666 ± 3.143	51.369 ± 3.044	51.125 ± 2.952	50.956 ± 2.903	50.924 ± 2.881
119(p)	53.9	53.4	53.2	52.2	51.4	51	50.8	50.5	50.3	50.7	49.8	49.1
119(model)	53.95 ± 1.32	53.386 ± 1.244	52.79 ± 1.179	52.168 ± 1.149	51.69 ± 1.1	51.235 ± 1.068	50.871 ± 1.036	50.523 ± 1.001	50.245 ± 0.969	50.019 ± 0.94	49.868 ± 0.966	49.853 ± 0.941
120(p)	56.8	56	55.5	54.7	54	53.4	53	52.3	52.1	51.3	51.2	51.6
120(model)	57.099 ± 1.013	56.485 ± 0.975	55.839 ± 0.973	55.168 ± 0.949	54.641 ± 0.947	54.14 ± 0.975	53.729 ± 1.017	53.335 ± 1.051	53.012 ± 1.139	52.741 ± 1.188	52.547 ± 1.335	52.489 ± 1.436
129(p)	55.8	55.5	54.8	53.8	53.4	53.4	53.3	53.2	53	52.8	52.8	52.2
129(model)	55.524 ± 3.382	54.936 ± 3.191	54.314 ± 3.045	53.668 ± 2.915	53.165 ± 2.79	52.687 ± 2.681	52.3 ± 2.61	51.929 ± 2.572	51.628 ± 2.567	51.38 ± 2.575	51.207 ± 2.587	51.171 ± 2.62
130(p)	56.9	56.6	56.6	55.4	55.7	55.7	55.7	55.6	54.2	53.9	54.5	53.6
130(model)	56.902 ± 4.128	56.291 ± 3.888	55.648 ± 3.691	54.981 ± 3.561	54.457 ± 3.416	53.958 ± 3.349	53.55 ± 3.356	53.16 ± 3.422	52.839 ± 3.525	52.571 ± 3.48	52.379 ± 3.437	52.325 ± 3.48
126(p)	68.1	67.4	67.6	66	65.7	65.4	65.3	63	62	62.6	61.6	60.6
126(model)	68.316 ± 1.859	67.522 ± 1.757	66.699 ± 1.667	65.855 ± 1.677	65.156 ± 1.607	64.486 ± 1.571	63.91 ± 1.589	63.353 ± 1.691	62.869 ± 1.646	62.441 ± 1.649	62.09 ± 1.604	61.88 ± 1.576
127(p)	51.8	51.3	51.2	50.8	50.4	49.6	49.2	48.7	49.2	48.7	49.2	49.3
127(model)	51.982 ± 1.518	51.45 ± 1.435	50.885 ± 1.364	50.294 ± 1.312	49.845 ± 1.289	49.42 ± 1.274	49.085 ± 1.231	48.766 ± 1.19	48.515 ± 1.152	48.317 ± 1.165	48.193 ± 1.145	48.206 ± 1.205
22(p)	53.4	53	52.4	51.2	51.2	51.3	50.6	50.5	50	49.4	49.9	49.4
22(model)	53.261 ± 1.182	52.709 ± 1.117	52.123 ± 1.074	51.512 ± 1.037	51.044 ± 1.008	50.6 ± 0.971	50.246 ± 1.006	49.908 ± 0.988	49.639 ± 1.0	49.423 ± 0.985	49.282 ± 0.957	49.277 ± 0.973
23(p)	55	54.6	53.9	53.4	52.8	52.7	52.2	51.8	51.5	51.4	51.4	51.4
23(model)	55.229 ± 1.538	54.645 ± 1.457	54.029 ± 1.381	53.387 ± 1.318	52.889 ± 1.261	52.415 ± 1.211	52.032 ± 1.176	51.666 ± 1.139	51.369 ± 1.104	51.125 ± 1.073	50.956 ± 1.05	50.924 ± 1.041
19(p)	56.8	56.1	55.7	54.3	54	51.5	51.4	51.4	52.5	52.4	52.2	50.4
19(model)	56.902 ± 1.893	56.291 ± 1.784	55.648 ± 1.696	54.981 ± 1.615	54.457 ± 1.594	53.958 ± 1.551	53.55 ± 1.982	53.16 ± 2.207	52.839 ± 2.307	52.571 ± 2.243	52.379 ± 2.18	52.325 ± 2.123
20(p)	53	52.8	52.1	50.8	49.8	49.4	49.2	48.4	47.8	48.1	48.2	48
20(model)	53.36 ± 1.413	52.805 ± 1.353	52.218 ± 1.282	51.606 ± 1.223	51.136 ± 1.258	50.691 ± 1.414	50.335 ± 1.524	49.996 ± 1.582	49.726 ± 1.723	49.508 ± 1.909	49.365 ± 1.968	49.359 ± 1.986
29(p)	55.2	55.5	54.1	53.3	54.2	54	52.7	52.3	53	54.2	53.8	53.5
29(model)	53.95 ± 5.381	53.386 ± 5.136	52.79 ± 5.047	52.168 ± 4.87	51.69 ± 4.705	51.235 ± 4.722	50.871 ± 4.779	50.523 ± 4.709	50.245 ± 4.641	50.019 ± 4.69	49.868 ± 4.956	49.853 ± 5.142
30(p)	57	56.2	56	54.8	54.5	54.2	53.6	53.8	53.9	53.1	53.2	52.4
30(model)	57 ± 2.118	56.388 ± 1.995	55.743 ± 1.895	55.074 ± 1.811	54.549 ± 1.74	54.049 ± 1.67	53.639 ± 1.611	53.247 ± 1.556	52.925 ± 1.53	52.656 ± 1.556	52.463 ± 1.525	52.407 ± 1.522
26(p)	57.7	58	56.7	56.5	56	57.2	55.8	55.4	55.4	54.7	53.2	53.1
26(model)	57.492 ± 1.308	56.872 ± 1.24	56.22 ± 1.373	55.543 ± 1.339	55.01 ± 1.394	54.503 ± 1.444	54.086 ± 1.995	53.687 ± 2.116	53.358 ± 2.217	53.082 ± 2.362	52.881 ± 2.416	52.819 ± 2.355
27(p)	60.3	60	58.8	58.1	57.6	57.4	56.8	56.1	55.8	55.3	55.6	55.7
27(model)	60.149 ± 2.482	59.486 ± 2.34	58.792 ± 2.241	58.074 ± 2.135	57.501 ± 2.043	56.953 ± 1.961	56.497 ± 1.904	56.059 ± 1.845	55.692 ± 1.786	55.379 ± 1.732	55.142 ± 1.683	55.043 ± 1.651
4(p)	50	49.4	48.6	47.9	48.3	48.3	47.5	47.3	47.1	47.1	47.3	47.4
4(model)	49.916 ± 1.246	49.417 ± 1.175	48.884 ± 1.114	48.325 ± 1.074	47.908 ± 1.056	47.514 ± 1.037	47.209 ± 1.082	46.92 ± 1.055	46.7 ± 1.038	46.531 ± 1.025	46.435 ± 1.03	46.476 ± 1.077
5(p)	63.7	62.8	62.3	60.7	60	59.6	58.8	58.6	58.1	57.8	57.2	57.1
5(model)	63.888 ± 2.476	63.165 ± 2.336	62.412 ± 2.226	61.636 ± 2.121	61.006 ± 2.099	60.402 ± 2.09	59.891 ± 2.057	59.399 ± 2.063	58.978 ± 2.036	58.612 ± 2.018	58.323 ± 1.997	58.173 ± 2.009
1(p)	64.4	62.6	60.6	59.2	58.8	57.7	57.3	56.6	56.5	56.6	58.2	57
1(model)	64.183 ± 2.634	63.456 ± 2.485	62.698 ± 2.416	61.917 ± 2.622	61.282 ± 2.953	60.674 ± 3.146	60.159 ± 3.416	59.662 ± 3.608	59.237 ± 3.807	58.867 ± 3.918	58.574 ± 3.95	58.42 ± 3.848
2(p)	58.8	58.2	58.2	57.1	56.5	56.2	54.6	55.1	55.2	57.2	57.9	55.9
2(model)	59.46 ± 0.854	58.808 ± 0.916	58.125 ± 0.948	57.418 ± 0.904	56.855 ± 0.884	56.318 ± 0.871	55.872 ± 0.841	55.444 ± 1.041	55.087 ± 1.022	54.783 ± 0.992	54.556 ± 1.483	54.466 ± 2.094
11(p)	54.1	53.5	53.2	52.5	51.8	51.9	50.8	50.3	50	49.5	49.9	49.6
11(model)	53.852 ± 0.897	53.29 ± 0.861	52.695 ± 0.827	52.075 ± 0.844	51.597 ± 0.843	51.144 ± 0.817	50.782 ± 0.883	50.435 ± 0.853	50.158 ± 0.828	49.934 ± 0.806	49.784 ± 0.809	49.771 ± 0.789
12(p)	67.6	67.8	67.7	66.2	65.2	65.1	64.9	63.8	62.9	61.8	61.6	63.2
12(model)	67.922 ± 1.654	67.135 ± 1.573	66.318 ± 1.548	65.48 ± 1.691	64.787 ± 1.67	64.123 ± 1.619	63.552 ± 1.643	63.001 ± 1.73	62.523 ± 1.72	62.1 ± 1.678	61.755 ± 1.636	61.551 ± 1.593
8(p)	50.7	50.2	50.3	50.5	49.9	48.8	47.8	47.7	48.8	48.9	48.3	47.5
8(model)	53.36 ± 4.876	52.805 ± 4.92	52.218 ± 4.943	51.606 ± 4.846	51.136 ± 4.68	50.691 ± 4.543	50.335 ± 4.49	49.996 ± 4.525	49.726 ± 4.524	49.508 ± 4.409	49.365 ± 4.292	49.359 ± 4.205
9(p)	59.6	59.9	59.6	58	57.3	57.6	57.1	57.5	57.4	57.2	56.6	58.1
9(model)	59.755 ± 3.082	59.099 ± 2.905	58.411 ± 2.799	57.699 ± 2.759	57.132 ± 2.646	56.59 ± 2.541	56.14 ± 2.506	55.708 ± 2.469	55.346 ± 2.548	55.039 ± 2.661	54.807 ± 2.774	54.714 ± 2.82
104(p)	55.8	54.9	53.8	52.9	52.6	52.3	51.8	51.4	51.1	51.4	51.4	51.6
104(model)	56.016 ± 1.381	55.42 ± 1.308	54.791 ± 1.282	54.137 ± 1.358	53.627 ± 1.48	53.141 ± 1.529	52.746 ± 1.539	52.369 ± 1.563	52.061 ± 1.587	51.806 ± 1.606	51.626 ± 1.572	51.583 ± 1.533
105(p)	55.7	55	54.7	53.5	52.7	52.6	51.4	50.8	51.5	51.4	51.8	51.8
105(model)	55.918 ± 1.544	55.323 ± 1.462	54.695 ± 1.401	54.043 ± 1.334	53.534 ± 1.314	53.05 ± 1.342	52.657 ± 1.314	52.281 ± 1.423	51.974 ± 1.56	51.721 ± 1.53	51.542 ± 1.493	51.501 ± 1.458
101(p)	53.1	53.3	53.1	51.9	51.6	51.8	51.7	51.4	49.3	48.4	48.4	49.7
101(model)	53.064 ± 2.0	52.515 ± 1.884	51.933 ± 1.853	51.325 ± 1.898	50.859 ± 1.846	50.418 ± 1.818	50.067 ± 1.898	49.732 ± 2.015	49.467 ± 2.117	49.253 ± 2.054	49.114 ± 2.035	49.112 ± 2.007
102(p)	55.5	54.5	55.2	53.7	53.4	53.6	53	52.8	53	51.9	51.8	51.9
102(model)	55.721 ± 1.479	55.129 ± 1.401	54.505 ± 1.385	53.856 ± 1.383	53.35 ± 1.327	52.869 ± 1.274	52.478 ± 1.287	52.105 ± 1.271	51.801 ± 1.277	51.55 ± 1.365	51.374 ± 1.336	51.336 ± 1.315
111(p)	51.8	51.3	50.9	50.4	50.1	50.1	49.8	49.5	49.4	49.1	49	49.1
111(model)	51.588 ± 0.896	51.063 ± 0.855	50.504 ± 0.824	49.919 ± 0.82	49.476 ± 0.832	49.057 ± 0.869	48.728 ± 1.003	48.414 ± 1.113	48.17 ± 1.204	47.977 ± 1.308	47.858 ± 1.375	47.876 ± 1.435
112(p)	50.4	50.7	50.2	48.9	49	50.3	50.6	50.9	50.9	50.4	49	49
112(model)	50.014 ± 1.162	49.514 ± 1.124	48.979 ± 1.3	48.419 ± 1.438	48 ± 1.404	47.605 ± 1.455	47.299 ± 2.001	47.008 ± 2.567	46.786 ± 3.144	46.616 ± 3.632	46.519 ± 3.945	46.558 ± 4.001
108(p)	56.5	55.8	55.7	55.5	55	55.1	54.4	54.6	55.1	56.6	57.1	54.1
108(model)	56.804 ± 4.93	56.194 ± 4.648	55.553 ± 4.413	54.887 ± 4.204	54.364 ± 4.038	53.867 ± 3.891	53.461 ± 3.805	53.072 ± 3.705	52.752 ± 3.664	52.486 ± 3.728	52.295 ± 4.098	52.242 ± 4.545
109(p)	64.8	64.5	63.7	62.2	61.4	61.5	60.8	60.2	59.6	58.9	59.2	59.5
109(model)	64.675 ± 0.839	63.94 ± 0.795	63.174 ± 0.832	62.386 ± 0.852	61.744 ± 0.823	61.128 ± 0.812	60.605 ± 0.807	60.102 ± 0.785	59.669 ± 0.761	59.293 ± 0.739	58.993 ± 0.741	58.832 ± 0.727

*Note: 1)122(p) is actual experimental body weight value of subject 122, 122(model) is model body weight confidence interval of subject 122,* etc.

*2) In all 384 confidence intervals, 26 actual body weight values are outside the confidence interval, but 9 values from these 26 values are within the area of statistical handling error. So the unsatisfied rate of estimation is from 4.4% to 6.77%. It shows our model estimation is acceptable. 3)α is 5%. The confidence interval: estimated body weight ±*$t_{\alpha /2}(n-2)*SE$*, where SE is standard error, degree of freedom is n-2.*

## Competing interests

The authors declare that they have no competing interests.

## Authors’ contributions

ZG (Zhejun Gong) conceived the idea and wrote the manuscript. ZG (Zhefeng Gong) collected the experimental data. All authors read and approved the final manuscript.

## Reviewer’s report

**Reviewer 1(Dr. E. Cabral Balreira)**

The authors propose an interesting model between Energy intake and body weight based on Fick’s second law of diffusion. This is an important are of research and the present article provides an interesting contribution to this research area and can provide new insight into the body weight mechanisms. The work justifying the use of Fick’s second law of diffusion went to a substantial justification and it is well motivated and explained. The referee agrees with the authors that there should be an investigation of the model.

The referee recommends that a major revision of the paper is required in order to be published in the Biology Direct.

The main issues are outlined as follows.

· In the results section, the authors did not fully disclosed their hypotheses that the energy density of body mass is independent of time. They needed this fact to arrive at their model equation (4). This is not supported by the previous discussion and it is big hypotheses that needs more explanation.

Author reply: *Generally, for adult men (20-33y) in the Minnesota human starvation study, the change in body weight is largely due to fat mass (FM), but not fat-free mass(FFM). As we can see from Kyle* et al.*, fat-free mass does not change much at middle age (from 18-34y to 35-59y), especially when compared with fat mass which changes significantly during the same period* [17]. Considering that the energy density of FM is much higher than that of FFM, the energy change is largely decided by change in FM. Thus, the change in energy intake, d(ρ*V), is approximately the change in energy in fat mass, which can be represented as p*d(V), in which p is the energy density of fat mass. The energy density of fat mass is supposed to be a constant, so we think the formula d(ρ*V) = p*dV is valid and the possible error here won’t affect our conclusion significantly.

· In the section titled simulation of body weight change using the developed model, it is unclear how the authors obtained the experimental data.

*Author reply: Total Energy Expenditure (TEE) includes two major parts: Resting Energy Expenditure (REE), the amount of calories needed to maintain basic body systems and body temperature at rest; Activity Energy Expenditure (AEE), the amount of calories used during activity* [20]*. Net energy intake is the difference between food intake and TEE. Although TEE was not measured in the Minnesota starvation study, TEE can be obtained through calculating REE and AEE* [19,21]*.*

*REE is calculated from Basal oxygen (cc/min) and kcalorie equivalent per cc/min. The daily energy expenditure at rest converts cc of oxygen/min into liters of oxygen/day, multiplied by the kcalorie equivalent of oxygen. The caloric equivalent of each cc of oxygen consumed in the resting state is calculated on the basis of Thorne Martin Carpenter’s 1921 table *[19]*. The group’s REE of 994.2 kcal/day at S24 equals group oxygen consumption of 139.1 cc/min multiplied by 1.44 (1440 min divided by 1000) and the groups’ caloric equivalent of oxygen of 4.964 kcal/cc.*

We here give an example to show how AEE is calculated. 22 miles per week of outdoor walking means 3.14 miles walking per day. A man’s normal walking speed is 3 miles per hour or so. When a 54 kg man walks with speed of 3 mph, the energy expenditure is 3.6 kcal/min. At S24, the group’s body weight is 52.57 kg. The group’s energy expenditure is (52.57/54)*(3.14/3)*3.6*60 = 220.1(kcal/day) [21]. When a 54 kg subject walks at 3.5 mph for half hour per week on a treadmill, his energy expenditure is 4.2 kcal/min. The group’s energy expenditure is (52.57/54)*4.2*30/7 = 17.52 (kcal/day) [21]*. These two parts of walking energy expenditure added, we can know AEE is 237.62 kcal/day.*

So at S24, TEE is 1231.83 kcal/day, net energy intake is 409.8 kcal/day.

From their work, in page 8 below equation (9), the authors state that they simply generated data from their own model and use that same data to validate the model. Such approach is circular and does not support the model validation. It simply shows that the ISCEM algorithm is working properly.

The authors must validate their model using the actual experimental data which they display in Table 1. Using the data from the Minnesota human starvation study, the authors need to estimate the parameters of their model, plot the actual results against the model predictions and report the R^2^ value.

Author reply: *In fact, we* ac*tually estimated the model parameters using the experimental data from Table*1*. We actually used the experimental data from Table*1*to validate the model. We also plotted the actual experimental results against the model predictions and reported the R*^*2*^*value.*

· Finally, the authors need to better explain how the ISCEM algorithm works and how is the SCEM-UA algorithm optimizing the parameters in their nonlinear problem.

Author reply: *Corrected*.

**Reviewer 2(Prof. Yang Kuang)**

This paper address an interesting but potentially controversial modeling problem that due to the quality or simplicity of the data, may be modeled by other simple or simpler models. There seems to be no real difficulties in fitting the data sets used in the three Figures. For example, using the first few weeks' data, we can find a energy and mass conversion rate for each subject and then use their weekly Total Energy Expenditure (TEE) to predict their weekly weight. Maybe the authors can comment on why such a simple and intuitive approach was not explored?

Author reply: *We proposed a molecular diffusion based model to uncover the relationship between energy intake and body weight. We used the data from the Minnesota human starvation study to verify the validity of our molecular diffusion based model. Because the relationship between body weight and energy intake is not linear, to predict body weight simply using the energy and mass conversion rate is not feasible, even if from a pure data fitting purpose.*

**Reviewer 3(Dr. Chao Chen)**

The authors propose a mathematical model in which body weight at time t is a function of linear combination of an error function, erf(#/#t) (a monotonic increasing function), and its complement 1-erf(#/#t)(a monotonic decreasing function), derived from the hypothesis of molecular diffusion following Fick’s second law. The model is found to have a good fit to a set of data taken from the Minnesota human starvation study. However, only data from the second phase of the study during the 24 weeks starvation period are used for model fitting; excluding data of the control and recovery phases from the same study.

Author reply: *In order to make clear how the body weight is affected by energy intake, we chose the data of starvation period from the Minnesota human starvation study*.

The authors claim: “This model provides valuable insights into the neural basis of behavioral decisions and their resulting effects”. It is difficult to see, on the basis of the presentation, any mechanistic connection as claimed. This article is just a data fitting exercise because similar models that are linear combination of two monotonic functions of opposing trends can also adequately fit the data.

Author reply: *This sentence, “This model provides valuable insights into the neural basis of behavioral decisions and their resulting effects”, is deleted.*

We considered that molecular diffusion (of, for example, neuropeptides) plays an important role in body weight changes. Because molecular diffusion is accompanied by energy transference, we then describe the molecular diffusion based process with energy diffusion.

*Our purpose is not to do data fitting exercise, but to use the data from the Minnesota human starvation study to verify the validity of our molecular diffusion based model*.

Furthermore, this data fitting exercise leaves a lot to be desired: e.g., only the mean body weight over time were analyzed, as presented in Figures 1– 3; no body weight changes from individual’s baseline was analyzed; and no statistical analysis, such as confidence intervals, for predicted body weight changes were provided.

Author reply: *Please see Appendix A and Appendix B*.

Editorial issues:

Pages 7–8. Something must be wrong: it is unlikely that parameters are estimated to be identical when different data sets from S1-S24 and S1–S12 are used.

Author reply: *Corrected*.

First line on top of p9: “are” should be deleted.

Author reply: *Corrected*.
